# Impact of screw tip design on screw anchorage: mechanical testing and numerical simulation

**DOI:** 10.1186/s13018-024-04841-6

**Published:** 2024-07-30

**Authors:** Shiming Xie, Liqiang Cui, Jinhui Liu, Peidong Qing, Jingchi Li

**Affiliations:** 1 Department of Spine surgery, Mianyang Orthopedic Hospital, Mianyang, Sichuan Province 621052 P.R. China; 2 Department of Orthopedics, Luzhou Key Laboratory of Orthopedic Disorders, The Affiliated Traditional Chinese Medicine Hospital, Southwest Medical University, Luzhou, Sichuan Province 646000 P.R. China; 3 Sichuan Provincial Laboratory of Orthopaedic Engineering, Department of Bone and Joint Surgery, Affiliated Hospital of Southwest Medical University, Luzhou, Sichuan Province PR China

**Keywords:** Pedicle screw, Screw tip design, Bone-screw interfaces, Screw loosening, Screw anchorage ability

## Abstract

**Background:**

Screw loosening is a commonly reported issue following spinal screw fixation and can lead to various complications. The initial cause of screw loosening is biomechanical deterioration. Previous studies have demonstrated that modifications in screw design can impact the local biomechanical environment, specifically the stress distribution on bone-screw interfaces. There are several different designs of screw tips available for clinically used pedicle screws; however, it remains unclear whether these variations affect the local stress distribution and subsequent screw anchorage ability.

**Methods:**

This study conducted comprehensive biomechanical research using polyurethane foam mechanical tests and corresponding numerical simulations to investigate this topic. Models of pedicle screw-fixed osteoporotic polyurethane foam were created with two different clinically used screw tip designs (flat and steep) featuring varying tip lengths, taper angles, and diameters, as well as identical flank overlap areas and thread designs. The anchorage ability of the different models was assessed through toggle and pull-out test. Additionally, numerical mechanical models were utilized to compute the stress distributions at the screw and bone-screw interfaces in the different models.

**Results:**

Mechanical tests revealed superior anchorage ability in models utilizing flat-tipped screws. Furthermore, numerical modeling indicated improved anchorage ability and reduced stress concentration tendency in these models.

**Conclusion:**

Changes in screw tip design can significantly impact the biomechanical anchoring capability of screws. Specifically, flatter tip pedicle screws may mitigate the risk of screw loosening by alleviating stress concentration on bone-screw interfaces.

## Background

Stability is a crucial factor in spine surgery [1, 2]. The pedicle screw fixation system is the most commonly utilized spinal fixation device. In comparison to other methods of fixation, this device can effectively stabilize all three columns and has been extensively employed in the treatment of spinal degeneration, trauma, deformity, and tumors over the past twenty years [3, 4]. Screw loosening is a frequently observed complication for patients with pedicle screw fixation and poses a significant risk to fixational stability leading to subsequent issues. With an increasing number of pedicle screw operations and a rising prevalence of osteoporosis (the primary risk factor for screw loosening), there has been a progressive increase in the incidence of this complication, resulting in significant social and economic burdens [5, 6].

The potential for screw loosening has been widely investigated. Biomechanical deterioration plays a main role in the pathological process of screw loosening [7, 8]. Specifically, stress concentration at the bone-screw interface can trigger microfractures of bony structures surrounding the screw trajectory [9, 10]. Resulting bony compaction and space progression around the screw trajectory can trigger the loss of screw anchorage ability, especially in osteoporotic patients with poor bony strength. The effect of screw design changes (e.g., thread design and screw outlines) on the risk of screw loosening and its biomechanical mechanism have also been widely investigated [11, 12]: Changes in screw design can affect local stress distribution pattern, and resulting risk of screw loosening [13, 14], the interaction between screw design changes and other fixation devices can also affect the spinal fixation stability [7, 15].

As an important design parameter, the design of the screw tip varies significantly among different clinically used pedicle screws [16, 17]. Generally, screw tips can be categorized into “flat” and “steep” design protocols, which are reflected by the screw tip length, tip diameter, and taper angle [4, 18]. Changes in screw length, diameter, and outline angle can impact anchorage ability by influencing the local biomechanical environment. Therefore, this study hypothesize that changes in screw tip design can biomechanically affect the screw anchorage ability. The main objective of this study was to validate this hypothesis and provide theoretical guidance for optimizing screw design. To address this topic comprehensively, our study conducted mechanical tests and numerical simulations. To our knowledge, this is the first study to explore this topic.

## Materials and methods

### Mechanical tests on the osteoporotic polyurethane foams

#### Model construction

**Fig. 1:**
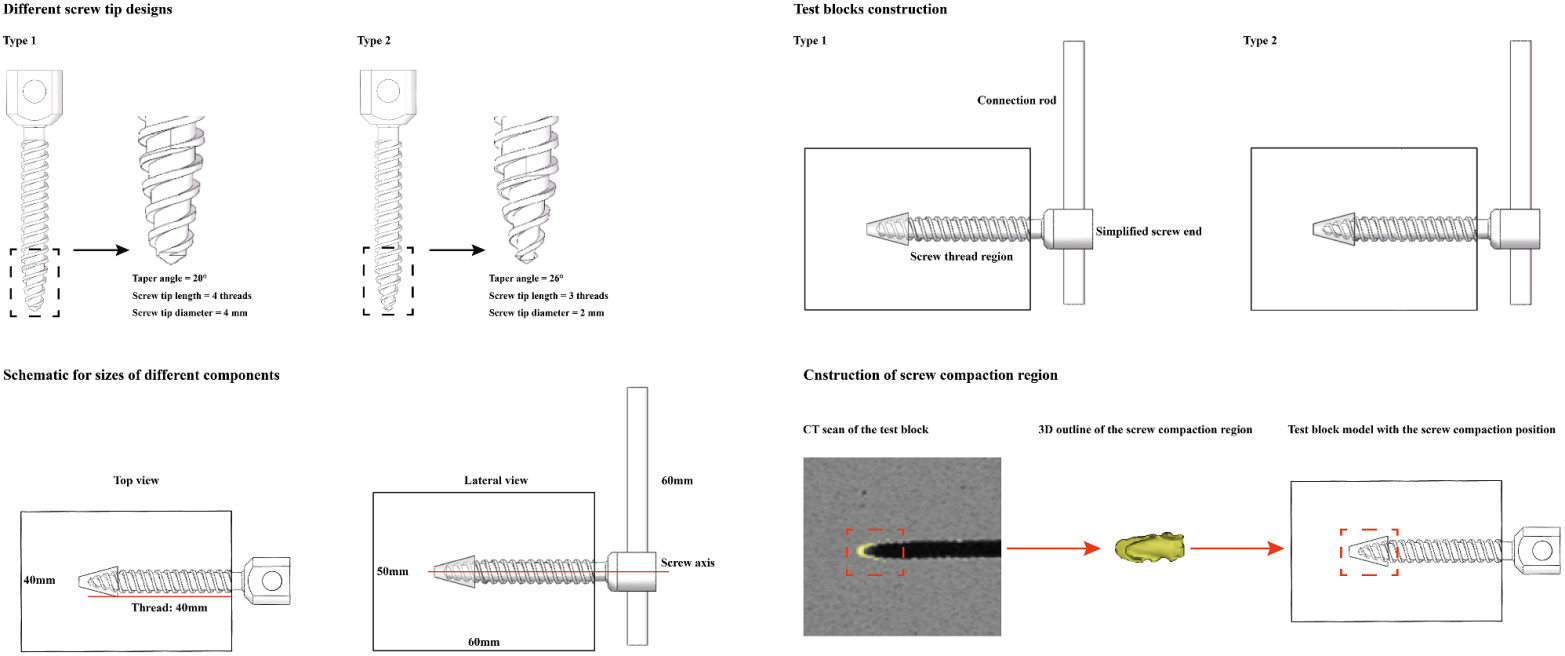
Different screw tip design types, assembly of the tested models, schematic for sizes of different components used in the current biomechanical research, and the CT scan based bony compaction region construction

Osteoporotic polyurethane foams (Sawbones Company, USA) have been used as a substitute bony material due to their homogenous structure, consistent material properties, and availability [19, 20]. Given that screw loosening is commonly observed in osteoporotic patients, the polyurethane foam density was selected to be 0.16 g/cm3 according to the standard from the American Society of Testing Materials (ASTM) protocol. The polyurethane foam was cut to 60 mm in length, 40 mm in width, and 50 mm in height. Two different cylindrical titanium alloy (Ti-6Al-4 V) pedicle screw types were selected for this study (WSD Company, China). The screw diameter and thread region length were set to 6.5 mm and 40 mm, respectively. These diameters are the most commonly used in our clinical practice (Fig. 1). The orientation of the sawbones test blocks was consistent throughout the processes of screw insertion, toggle, and pull-out load application.

To investigate the biomechanical significance of screw tip design changes, three-dimensional models of two different screw tips were constructed by changing the tip diameter, tip length, and taper angle. The detailed model construction strategy is presented in Fig. 1. The other design parameters of these two types of screws are identical. The volumes of the screws with flat and steep tips were completely identical, and the flank overlap area (FOA) in the models with different groups of screws was also identical [21, 22]. This approach allowed for the elimination of confounding effects stemming from other design parameters. The two screws were machined separately and inserted into the test block with a 40 mm insertion length (threads were completely inserted into the test blocks). The screw trajectory was coaxial to the central axis of the test block.

**Fig. 2:**
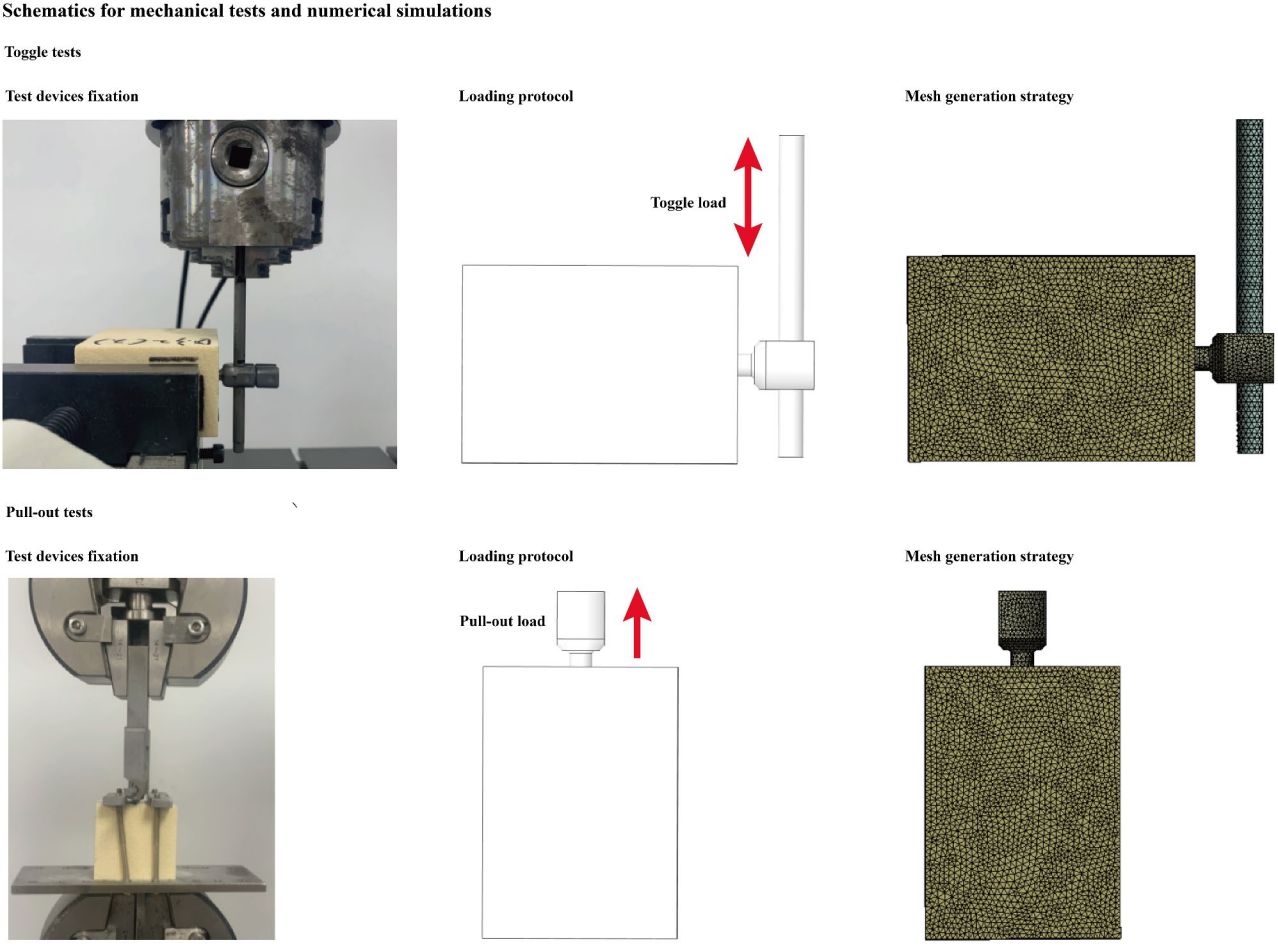
Tested device fixation, loading application protocol, and mesh generation strategy during mechanical tests and numerical mechanical simulations

**Fig. 3:**
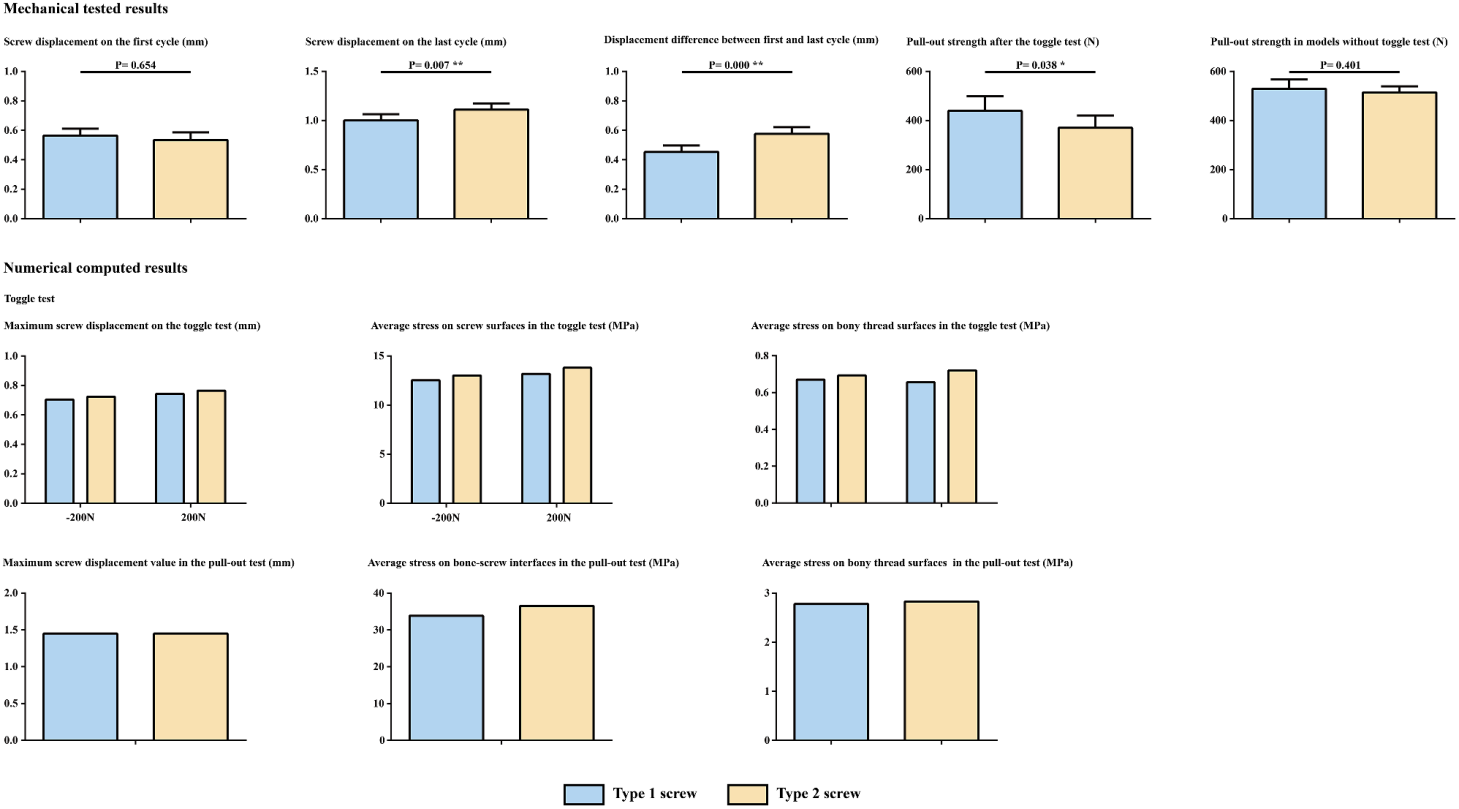
Tested and computed results for models with different screw types. Based on these results, this study present that the pedicle screw with flat tip design has better anchorage ability and lower risk of screw loosening

**Fig. 4:**
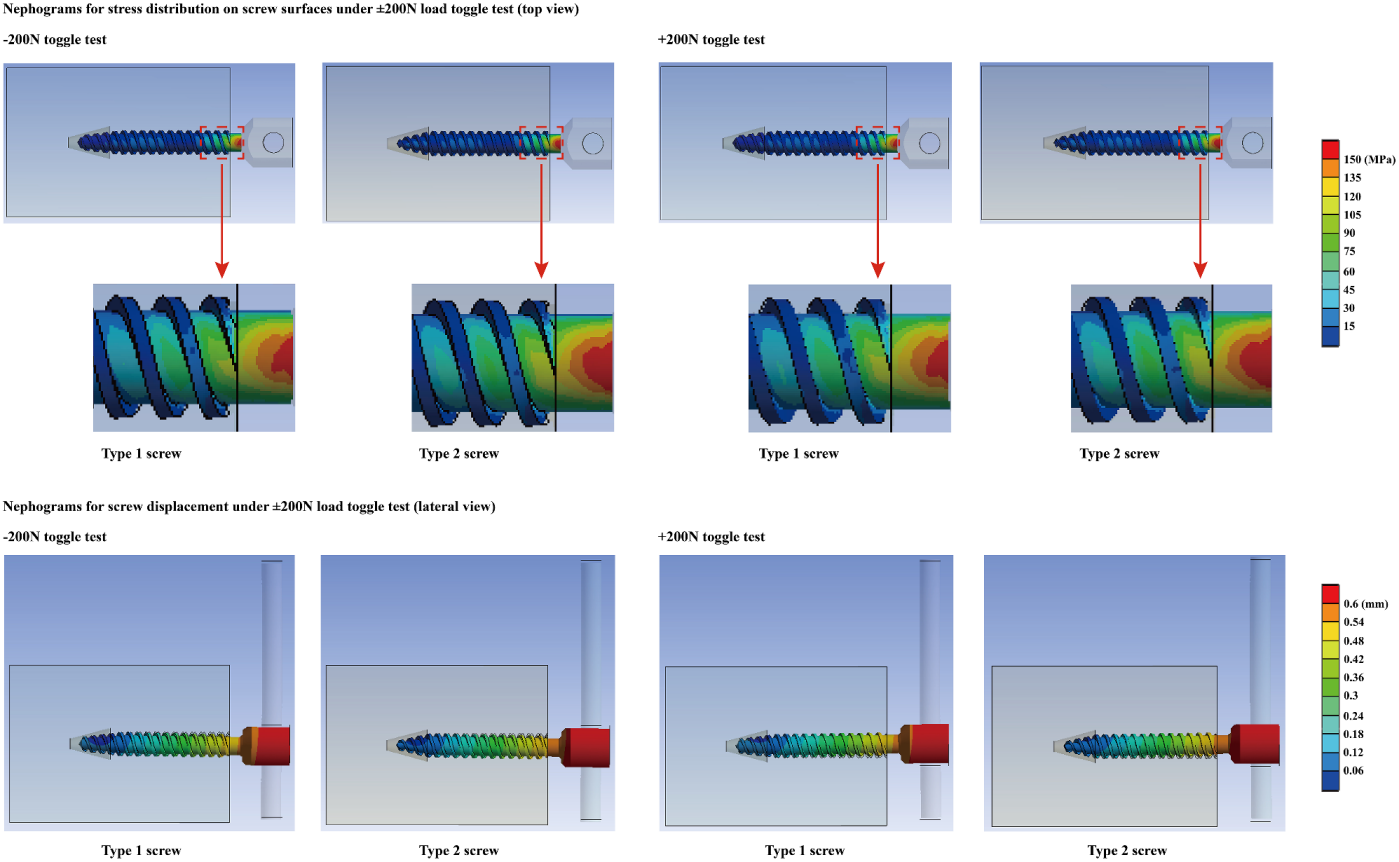
Nephograms for stress distribution on bone-screw interfaces and displacement of screw during the simulation of toggle tests

#### Toggle tests under different loading protocols

Toggle and pull-out tests were performed in an E3000 fatigue testing machine (Instron Company, USA). Each single test was repeated ten times for the different models. Each screw, connection rod, or nut was tested only once. Before the toggle tests, a connection rod (with an outer diameter of 6.0 mm and length of 100 mm) was inserted into the screw tulip and fixed with nails. The axis of the rod was vertical to that of the pedicle screw, and the distance from the screw axis to the rod tip was set to 60 mm. In the toggle tests, foam blocks were fixed in the testing machine. Each group was tested ten times in toggle tests. Toggle tests were performed under a constant load of 1*10^4^ cycles (screw loosening is a common early postoperative complication, and this cycle number adequately represents the load cycle during early postoperative activity), and the pedicle screw was subjected to cyclic loading in the cranio-caudal direction with a ± 200 N load (representing the single screw load during daily walking in a 40 kg osteoporosis patient) [23, 24]. Cyclic loading was terminated after 1 × 10^4^ loading cycles. The screw displacement values in the first and last cycles and the differences between these two displacement values were recorded in this procedure (Fig. 2) [25, 26].

#### Pull-out tests

Pull-out tests were performed in models with and without instant load toggle tests. In the pull-out tests, the foam was also strictly fixed to the testing machine, and a custom-made fixture connected to the testing machine was then attached to the connection rod. By this method, the axis of the screw was collinear to the pull-out force. All screws were pulled uniaxially at a rate of 5 mm/min until they were pulled entirely out of the foam, and the displacement value was measured 100 times per 1 s [3, 18]. The pull-out strength was judged as the axial force when a sudden decrease in the pull-out force was observed (Fig. 2) [27, 28].

#### Statistical analyses

Statistical analyses was accomplished in the SPSS 26.0 software. This study conducted single-sample K-S tests to verify the normality of each continuous variable. The results indicated that all variables had P-values > 0.05, indicating conformity to the normal distribution. Therefore, these parameters are presented as the mean ± standard deviation [29, 30]. Differences in these parameters between flat and steep screw groups were compared by independent sample t tests. Moreover, the correlation between parameters in tested in toggle tests and pull-out strength (in models with toggle tests) were determined by computing the pearson’s correlation coefficient. A P value less than 0.05 indicated a significant difference [31, 32].

### Numerical simulations

#### Numerical model construction

Pedicle screw models with different screw tip designs were developed using “UG NX10.0”. The models utilized in the mechanical tests were obtained from a screw processing company and were completely identical to the screws used in the aforementioned mechanical tests. Numerical simulations for this study were carried out using “Ansys Workbench 2020 R2 Academic”. The model construction strategy, boundaries, and loading conditions for numerically simulating toggle and pull-out tests are similar to those used in the mechanical tests. To enhance computational efficiency, simplifications were applied to the numerical models. Specifically, in the numerical simulations, the size of the test blocks matched that of the mechanical tests (60 mm*55 mm*40 mm), with an insertional screw depth set at 40 mm. The connection between the screw tulip, nut, and spacer was simplified into a single model [7, 33].

The axis of the screw was vertical to the connection rod, the distance between the screw axis and the rod tip was 60 mm, and the rod on the caudal side was deleted to reduce the element number. When defining the material properties of different components, test blocks were set according to the official product parameter table from the saw-bone company (Fig. 1). Moreover, the screw compaction (consolidation) effect caused by screw insertion has also been simulated by enhancing the material properties of the surrounding bony structure. The region of bony compaction was defined based on the CT scan of test blocks in published studies [33, 34]. The elastic modulus of bone was assumed to be a power-law function of the density with an exponent of 2 [34, 35]. The pedicle screw and connection rod were defined as titanium alloy material (elastic modulus = 12 GPa and Poisson’s ratio = 0.31) [7, 33], and the test block was defined based on the produce manual of osteoporotic polyurethane foams from the Sawbone Company (elastic modulus = 23 MPa and Poisson’s ratio = 0.3) (Fig. 1) [7, 33].

#### Simulation of toggle tests

To ensure computational credibility, the boundary and loading conditions of the numerical simulation were kept identical to those in the toggle test. The contact type between the screw and connection rod was defined as “bonded”, that between the bone–screw interfaces was defined as “frictional”, and the friction coefficient between these interfaces was 0.2. All freedom of the foam models was completely fixed, and a ± 200 N load in the cranial-caudal direction was applied to the tip of the connection rod [22, 33]. The mesh generation strategy was identical in the two groups of models. To eliminate the confounding effect caused by mesh size, this study performed a mesh convergence test. Different sizes of tetrahedron element was selected in this study (Table 1). By evaluating the change in the maximum equivalent stress on the pedicle screw, the mesh sizes on the screw and foam were adjusted. The model was considered converged if the change in the computed stress values on the test block was less than 3% [36, 37]. To represent the potential risk of screw loosening, the screw displacement value and the stress distribution on the bone-screw interfaces were computed and recorded in this study (Fig. 2).

#### Simulation of pull-out tests

The material property definitions, mesh sizes, and contact types on the bone-screw interfaces in the pull-out test were consistent with those in the toggle test. The construction of the test block and the screw models was also consistent with the toggle test, but the pulling-out test connecting rod was deleted to reduce the element number [38, 39]. The degrees of freedom of the test block were completely fixed, and a 500 N load along the axis of the pedicle screw was applied to the screw tulip. According to the results of the toggle test, the screw displacement and the stress distribution on the bone-screw interfaces were also computed (Fig. 2).

## Results

### Mechanical test results

Compared to the steep screw tip, the flat design results in better screw anchorage ability In the toggle test, there was no significant difference in screw displacement during the first cycle. However, the flat screw tip group showed significantly lower displacement of screws during the last cycle and a significant difference in displacement between the first and last cycles. Additionally, the pull-out strength was significantly greater in the flat screw tip group. Although there was no significant correlation between the screw pull-out strength after the toggle test and the first cycle screw displacement, a significant negative correlation was observed between the last cycle screw displacement, displacement difference, and pull-out strength (Fig. 3; Tables 2 and 3). Therefore, based on this study, compared to pedicle screws with a flat tip, screws with a steep tip are more likely to experience loosening during immediate postoperative period.

### Numerically simulated results

Compared to models fixed with steep-tip screws, flat-tip screws suffer greater screw displacement and greater stress on the bone-screw interfaces during the simulation of the toggle test. However, although the stress on the bone-screw interfaces was still greater in the steep-tip-screw fixed model in the pull-out test, the screw displacement remained identical among the different models under a 500 N axial pull-out load (Figs. 3 and 4; Table 4).

## Discussion

Screw loosening can lead to loss of fixation stability, resulting in various complications and deterioration of patient prognosis [40, 41]. Modifying screw design to optimize fixation stability has been shown to effectively improve patient clinical outcomes with pedicle screw fixation [42, 43]. The potential impact of screw design changes on screw anchorage ability has also been widely reported. Given the highly adjustable nature of screw tip design and its potential influence on screw loosening, this study hypothesized that changes in screw tip design may affect the local biomechanical environment and screw anchorage ability. To test this hypothesis, comprehensive biomechanical research was conducted including mechanical tests on osteoporotic polyurethane foam and corresponding numerical simulations. Results showed that compared to a steep screw tip design, a flat pedicle screw can achieve better anchorage ability by reducing stress concentration at the bone–screw interfaces. Based on mechanical testing and numerical computations, this study presents a feasible method for optimizing pedicle screw design which holds clinical significance for patients with fixed screws by reducing the potential risk of loosening and fixation failure.

The following topics need to be clarified. Firstly, when evaluating changes in screw tip design protocols, this study constructed two types of screws with different tip lengths, diameters, and taper angles. However, the current study did not separately discuss these three variables. Previous research has indicated that increasing screw volume and FOA (insertion angle) can effectively optimize the stability of pedicle screws [41, 44]. This change may explain why larger and longer screws can enhance screw anchorage ability. During the model construction process, it was found that only by simultaneously adjusting these three variables (tip diameter, tip length, and taper angle), could we ensure that the volume of the two screw types remained identical along with resulting FOA values for both flat and steep tips. Under such conditions, it can be shown that differences in local stress distribution and screw anchorage ability between the two groups were rooted in changes in screw tip design itself rather than in their volume or FOA [21, 22].

In this study, a contradiction is observed between the tested pull-out strength and the numerically computed screw displacement value under the pull-out load. The tested pull-out strength following the toggle cyclic load was found to be significantly lower in models with steep screw tips, while completely identical screw displacement values were recorded in the simulation of the pull-out load. Although the ability of pull-out strength to represent screw anchorage in models without toggle cyclic load has been widely tested, it is important to note that screw pull-out remains a relatively rare complication compared to microfracture around bony structures along screw trajectories and resulting screw loosening [44, 45]. This study infers that direct influence of screw design parameters on pulling strength is limited; however, the incidence of microfracturing along the screw trajectory under cyclic toggle loading can decrease the pull-out strength of pedicle screws. The correlation analysis results in this study confirm this finding: there is a significant correlation between pull-out strength after cyclic loading and screw anchorage parameters. Therefore, it cannot be denied that the role of a pull-out test in studies on validating screw anchorage ability is crucial based on this study’s findings. Accordingly, it is recommended to perform a pull-out test after cyclic loading rather than conducting a direct pull-out test. In other words, when evaluating fixation strength of pedicle screws, it should be considered that pull-out strength serves as an indicator during cyclic loading rather than being used independently [13, 40].

Furthermore, due to limitations in computational techniques, it is not possible to simulate the microfracturing of the screw trajectory and subsequent compaction of surrounding bony structures under cyclic loading in numerical mechanical simulations. These simulations can only record stress and displacement values for one load cycle. Despite this limitation, published studies have indicated that displacement and stress are reliable indicators of the risk of screw loosening during cyclic loading. This assertion has been corroborated by the present study which found that screws experiencing higher stress exhibited poorer anchorage ability during instant load toggle tests and pull-out tests following cyclic loading [9, 46]. Importantly, while mechanical tests can directly demonstrate changes in screw anchorage ability, they cannot fully explain the potential biomechanical mechanism behind such phenomena as they do not directly reflect detailed stress distribution patterns on bone-screw interfaces [42, 47].

Therefore, this study conducted comprehensive research involving mechanical tests and numerical simulations. The experimental approach combined two techniques to ensure consistency in entry parameters (e.g., model outlines, boundary and loading conditions) for both mechanical tests and numerical simulations. This method effectively overcomes the limitations of individual experiments and enhances the credibility of the experimental results. Previous studies have indicated that normal bone density models achieve optimal fixation strength regardless of screw design, while reduced bone density leading to poor bone strength is a primary cause of screw loosening. In line with commonly used research protocols in similar studies [48, 49], only osteoporotic models were utilized in this study.

To investigate the effect of screw tip design changes on screw anchorage ability separately, the screws used in this study were specially designed and machined. This method can eliminate the confounding effects caused by other design parameters, screw insertion volumes, and FOA values on the experimental results (these parameters are completely consistent between the experimental and control groups). However, custom-made screws cannot be directly used in clinical practice for the lack of authority, and the lack of clinical evidence was the main limitation of this study. Moreover, the influences of different screw design parameters on screw anchorage ability interact, and the biomechanical significance of screw tip design changes has only been validated for a single screw type. The influence of other factors (e.g., thread design) on the current research conclusion have also yet to be identified. We hope to obtain production authorization for the modified screw as soon as possible in future studies and validate the conclusions of this study through clinical studies. At the same time, through the systematic biomechanical research method as in this study, the interaction of screw tip design changes and other design parameters changes is further explained to further optimize the screw design scheme.

To investigate the impact of changes in screw tip design on screw anchorage ability, specially designed and machined screws (rather than commercially available designs) were used in this study. This approach allowed for the elimination of confounding effects stemming from other design parameters, screw insertion volumes, and FOA values on the experimental results - ensuring complete consistency between the experimental and control groups in terms of these parameters. However, it should be noted that custom-made screws cannot be directly utilized in clinical practice due to a lack of authorization, and the absence of clinical evidence represents a primary limitation of this study. Furthermore, it is important to recognize that different screw design parameters may interact with each other when influencing screw anchorage ability; thus far, only one type of screw has been biomechanically validated for its tip design changes. The potential influence of other factors (such as thread design) on our research conclusions remains unexplored at present. It is our hope to secure clinical use authorization for the modified screws as soon as possible in future studies and substantiate the findings presented here through clinical investigations. Concurrently, by employing a systematic biomechanical research method akin to that employed within this study, we aim to shed further light on how alterations in screw tip design interact with changes in other design parameters so as to advance our optimization efforts pertaining to screw designs.

## Conclusions

Through comprehensive biomechanical research involving clinical review and numerical simulations, this study has demonstrated that changes in the design of screw tips can have a significant biomechanical impact on their anchorage ability. Specifically, it was found that flatter tip pedicle screws may reduce the likelihood of screw loosening by reducing stress concentration at the bone-screw interfaces. However, it is important to note that further validation of these research findings in clinical practice is necessary. Additionally, our future studies will aim to clarify the interaction between screw tip design and other parameters of screw design.

**Table 1:** Mesh generation strategy of components used in this study

	Element size (mm)	Element number	Node number	Average element quality
**Test block**	1.2	695,728	503,678	0.853
**Pedicle screw**	1.0	32,167	20,156	0.831
**Connection rod**	1.25	11,309	6992	0.872
**Bony compaction region**	0.15	763,090	538,753	0.852

By using this mesh generation strategy, the element quality of each components was larger than 0.8, which effectively ensure the computational credibility of the current study

**Table 2:** Significant differences comparison of mechanical testes results

	Flat screw tip	Steep screw tip	*P*-values
**Screw displacement on the first cycle (mm)**	0.546 ± 0.047	0.534 ± 0.052	0.654
**Screw displacement on the last cycle (mm)**	1 ± 0.064	1.111 ± 0.063	0.007**
**Displacement difference between first and last cycles (mm)**	0.453 ± 0.044	0.576 ± 0.045	0.000**
**Pull-out strength after the toggle test (N)**	439.14 ± 59.94	370.29 ± 50.01	0.038*
**Pull-out strength in models without toggle test (N)**	529.14 ± 38.58	514 ± 25.11	0.401

* *P*<0.05; ** *P*<0.01

**Table 3:** Correlation coefficients between toggle test results and pull-out strength after toggle test

	Correlation coefficients	*P*-values
**Screw displacement on the first cycle (mm)**	-0.466	0.093
**Screw displacement on the last cycle (mm)**	-0.793	0.001**
**Displacement difference between first and last cycles (mm)**	-0.574	0.032*

* *P*<0.05; ** *P*<0.01

**Table 4:** Computed results during toggle and pull-out test simulations

	Flat screw tip	Steep screw tip
Toggle test (-200 *N*)		
Maximum screw displacement (mm)	0.705	0.742
Average stress on screw surfaces (MPa)	12.53	13.18
Average stress on bony thread surfaces (MPa)	0.67	0.694
**Toggle test (+ 200 N)**		
Maximum screw displacement (mm)	0.724	0.765
Average stress on screw surfaces (MPa)	13.02	13.8
Average stress on bony thread surfaces (MPa)	0.694	0.72
**Pull-out test (500 N)**		
Maximum screw displacement (mm)	1.45	1.45
Average stress on screw surfaces (MPa)	33.86	36.52
Average stress on bony thread surfaces (MPa)	2.78	2.828

## Data Availability

No datasets were generated or analysed during the current study.
